# Coping with Stress in Deprived Urban Neighborhoods: What Is the Role of Green Space According to Life Stage?

**DOI:** 10.3389/fpsyg.2017.01760

**Published:** 2017-10-18

**Authors:** Jenny J. Roe, Peter A. Aspinall, Catharine Ward Thompson

**Affiliations:** ^1^Center for Design and Health, School of Architecture, University of Virginia, Charlottesville, VA, United States; ^2^OPENspace Research Centre, University of Edinburgh, Edinburgh, United Kingdom

**Keywords:** latent class analysis, latent health cluster, health cluster membership, perceived stress, stress coping scenario, deprived urban neighborhood, green space quality

## Abstract

This study follows previous research showing how green space quantity and contact with nature (via access to gardens/allotments) helps mitigate stress in people living in deprived urban environments (Ward Thompson et al., 2016). However, little is known about *how* these environments aid stress mitigation nor how stress levels vary in a population experiencing higher than average stress. This study used Latent Class Analysis (LCA) to, first, identify latent health clusters in the same population (*n* = 406) and, second, to relate health cluster membership to variables of interest, including four hypothetical stress coping scenarios. Results showed a three-cluster model best fit the data, with membership to health clusters differentiated by age, perceived stress, general health, and subjective well-being. The clusters were labeled by the primary health outcome (i.e., perceived stress) and age group (1) *Low-stress Youth* characterized by ages 16–24; (2) *Low-stress Seniors* characterized by ages 65+ and (3) *High-stress Mid-Age* characterized by ages 25–44. Next, LCA identified that health membership was significantly related to four hypothetical stress coping scenarios set in people's current residential context: “*staying at home*” and three scenarios set outwith the home, “*seeking peace and quiet,” “going for a walk*” or “*seeking company.*” Stress coping in *Low stress Youth* is characterized by “*seeking company*” and “*going for a walk*”; stress coping in *Low-stress Seniors* and *High stress Mid-Age* is characterized by “*staying at home.*” Finally, LCA identified significant relationships between health cluster membership and a range of demographic, other individual and environmental variables including access to, use of and perceptions of local green space. Our study found that the opportunities in the immediate neighborhood for stress reduction vary by age. Stress coping in youth is likely supported by being social and keeping physically active outdoors, including local green space visits. By contrast, local green space appears not to support stress regulation in young-middle aged and older adults, who choose to stay at home. We conclude that it is important to understand the complexities of stress management and the opportunities offered by local green space for stress mitigation by age and other demographic variables, such as gender.

## Introduction

This study explores stress patterns amongst people living in poverty and how these patterns relate to potential stress coping behaviors. It builds on our earlier research which shows that higher levels of green space in the neighborhood environment are associated with lower stress as measured by perceived stress (Ward Thompson et al., 2016) and diurnal patterns of cortisol (Ward Thompson et al., 2012; Roe et al., 2013). We were particularly interested in the current study on the opportunities that green space can offer for initiating and supporting stress regulating activities. First we set out the rationale—and evidence—for exploring green space and neighborhood attributes in relation to stress regulation and then present our methods and results.

### Stress regulation, green space, and neighborhood attributes

Most studies exploring relationships between stress and the environment focus on negative relationships: many studies have identified the features of the neighborhood environment that are associated with poor mental health (e.g., depression, anxiety, mood disorders, poor cortisol regulation, reduced cognitive functioning) such as air and noise pollution, traffic levels, high density living, and crime and violence (Aneshensel and Sucoff, 1996; Hadley-Ives et al., 2000; Robinson and Keithley, 2000; Ross and Mirowsky, 2001; Latkin and Curry, 2003; Chu et al., 2004; Gee and Takeuchi, 2004; Powdthavee, 2005; Gary et al., 2007; Chaix et al., 2008; Echeverria et al., 2008).

Fewer studies, however, have focused on the environmental attributes that support or encourage opportunities for stress mitigation. Our previous study found beneficial relationships between perceived stress and the quantity of, and access to, green space (via gardens and allotments) in deprived urban communities (Ward Thompson et al., 2016). A Danish study found increases in perceived stress in individuals living more than 1 km away from a green space (Stigsdotter et al., 2010). In the USA, higher levels of neighborhood green space have been associated with significantly lower levels of perceived stress (Beyer et al., 2014).

There is also evidence that green space has a positive effect on stress physiology. A series of Japanese studies have shown the beneficial effects of walking in forests and natural environments on physiological stress, including cortisol levels, pulse rate, blood pressure and heart rate variability (Park et al., 2010; Toda et al., 2013). The quantity of green space has also been found to have a positive effect on physiological stress regulation—as measured by diurnal daily patterns of cortisol—in deprived urban Scottish communities (Ward Thompson et al., 2012; Roe et al., 2013). A further UK study found chronic stress, as measured by hair cortisol concentration, was higher in neighborhoods with less green space, but effects were attenuated beyond significance when controlling for income deprivation (Gidlow et al., 2016).

In addition, there is evidence to suggest green space can act as a buffer to everyday life stressors in urban and rural neighborhoods, as well as having a direct effect on stress physiology. The presence of green space within a 3 km radius of a resident's home has been shown to attenuate the negative health impacts of stressful life events in Dutch adults (van den Berg et al., 2010). Research in rural USA communities has shown that nature in the immediate vicinity of the residential environment may serve as a buffer for the impact of stressful life events on children's psychological well-being (Wells and Evans, 2003). In deprived urban neighborhoods in the USA, Kuo (2001) found residents living with more neighborhood green space were significantly better able to manage major life issues (as measured by manageability of personal goals) than those residents living in areas with less green space.

### The role of green space in stress regulation

Research has suggested that visiting favorite places helps emotional self-regulation, including stress relief (Korpela, 2003). Emotional self-regulation is defined as actively coping with moods and emotional situations; a person may employ psychological, physical, social or environmental strategies in order to regulate negative mood (Korpela, 2003). Typically, the environments people seek after the experience of a negative antecedent (e.g., stress, bad mood, a quarrel with someone) are favorite places which offer relief and opportunities for emotional self-regulation (Korpela and Ylén, 2007). Research has shown that natural environments rank highly as favorite places and also offer a context for emotional self-regulation (Korpela, 2003; Johnsen and Rydstedt, 2013). A central idea within this body of research is that natural places have restorative attributes—that, for example, they are inherently fascinating and offer a context for “being away” from everyday stressors (Kaplan and Kaplan, 1989)—which support emotional regulation and recovery from low mood, fatigue and stress (Korpela, 2003). This research suggests that the active and repeated use of natural spaces for ongoing emotional self-regulation can help support resilience over time (Korpela, 2012). Environmental emotional regulation strategies therefore hold much promise for supporting well-being, both in the short-term and long-term, but there is little empirical evidence showing how the natural environment affords, or contributes to, stress-regulation in deprived urban communities experiencing major life stressors. The affordances of the environment refers to the functional properties an environment affords an individual for action, described in terms of what is *do-able* (Heft, 1988).

Opportunities for contact with nature vary enormously across socio-economic and cultural contexts. In the UK, for instance, it is known that poorer urban communities live with less green space and poorer quality green space (CABE, 2010). Nature affordances are therefore affected by the inequalities in green space provision. The type of contact with nature also varies among people, e.g., viewing nature passively from a window, or being physically active in nature (e.g., walking or gardening), as does the frequency and duration of such contact with nature (Hartig et al., 2014). Climate, seasonality, the varying needs of population sub-groups (e.g., gender, age, and ethnicity), as well as culture and individual circumstance, will all impact on the experience of nature affordances by an individual. One of the aims of our study was to better understand the use of nature to afford stress reduction in different segments of deprived urban communities.

Our primary interest is in deprived urban neighborhoods since research suggests the association between green space and health—both for all cause mortality and for mental well-being–tends to be stronger in poorer communities (Mitchell and Popham, 2008; Mitchell et al., 2015). If access to, and use of, green space can be improved in deprived urban communities, current evidence suggests this may help address health inequalities (Allen and Balfour, 2014).

Our study used Latent Class Analysis (LCA) to, first, model clusters distinguished by general health and mental health outcomes in a sample of people aged 16 to 85 living with deprivation and, second, to explore the causes of health cluster membership and its relationship to a range of variables, including hypothetical environmental stress coping scenarios, individual circumstances and neighborhood environmental characteristics, including green space.

Four research questions guided our analysis. Amongst a sample of urban residents living in poverty:

RQ1: What different health clusters, as identified by latent class, can be found within these deprived communities?
RQ2: How are stress coping behaviors associated with the latent health clusters?
RQ3: How do demographic and other individual characteristics relate to the latent health clusters?
RQ4: How do environmental variables (particularly green space) relate to the latent health clusters?

## Methods

### Study design

This was a cross sectional study designed to understand the coping strategies of deprived urban communities in relation to stress regulation. It is one of a series of studies carried out as part of the GreenHealth project for the Scottish Government, exploring relationships between stress and green space using the study setting described below. The Final Report summarizing the project as a whole (James Hutton Institute, 2014) can be found at http://www.hutton.ac.uk/research/projects/green-health.

### Study setting

Two areas in Central Scotland were selected on the basis of, firstly, high indices of poverty using the Carstairs Index for population data in 2001 (Carstairs and Morris, 1991). Carstairs scores are an index of deprivation at ward level (i.e., a spatial unit defining electoral boundaries in the UK) based on an unweighted combination of four census variables: unemployment, overcrowding, car ownership and low social class. A higher score equates with higher deprivation, with a score of greater than 6 indicating “very deprived areas.” Four areas in two cities were chosen based on the Carstairs Index from the most recent census data available at the time of data collection (2001) together with an objective measure of green space quantity, derived from ward level Census Area Statistics (CAS), created by the Centre for Research on Environment Society and Health (CRESH)^1^ and available at the CRESH website (www.cresh.org.uk). The green space measure includes parks, woodlands, scrub and other natural environments. Our selection was based on achieving as wide as possible a variation in publicly accessible green space levels (i.e., excluding private gardens) whilst maintaining the high deprivation criteria and matching other environmental criteria. This reflects the fact that the areas selected for this study are characterized by social rented housing. The higher green space wards in our sample offered access to parks and informal urban green spaces, including shared community gardens. Those wards with lower levels of green space lacked access to green space, either in the wider community or immediate home environment. Further information on ward characteristics can be found in Ward Thompson et al. (2016). Note, the CAS ward level measure of green space quantity was used for case study selection only.

### Stress coping scenarios

The stress coping scenarios for the questionnaire were identified by prior qualitative data collection via four focus groups with residents (*n* = 29) in our sampling locations. Groups were of mixed gender (31% male, 69% women) and mixed age (ranging between 18 and 65 plus). This identified four coping behaviors for self-initiated stress regulation in one's current residential environment: “*staying at home*” or going to “*some other place*” outwith the home. The latter behavior was further categorized into three outdoor behaviors: “*take a walk and get some fresh air” (subsequently referred to as “going for a walk,” “seeking peace and quiet*” and “*seeking company*.”) These behaviors—together with insights on the social and environmental contexts for each behavior choice—were used to design the questionnaire described in section Survey Variables and Outcome Measures below. Further information on the qualitative analysis is provided in the Final Report for the Scottish Government (James Hutton Institute, 2014), see section 3.2.2 http://www.hutton.ac.uk/research/projects/green-health.

### Recruitment and sample size

Participants were recruited from each of the four areas using post-codes that met the criteria set out in section Study Setting, above. Each case study area had a total population of ~5,000. Given the exploratory nature of this research, there was no basis for determining research power and the sample size was therefore largely determined by the limit on resources available to the study. A stratified sampling methodology was used that matched proportions of the sample to census data proportions (based on the 2001 national census and deprivation indices derived from this) for each case study area, based on age, gender and the deprivation criteria described above. This ensured a consistent sample of individuals experiencing similar levels of economic hardship. The survey was undertaken in June 2010. As a check on possible gentrification that might have occurred between the 2001 census and our survey, a check on subsequently published 2011 deprivation indices indicates that deprivation had worsened over time in 3 out of 4 case study areas, with the deprivation levels remaining constant in the fourth.

### Data collection

A cross-sectional household questionnaire was developed and administered by a survey company, using a face-to-face, computer-assisted interview (CAPI). Prior to the survey, introductory letters were posted to residents in the sample area informing them about the survey. Recruitment was door-to-door by fieldworkers in four defined locations (as described in section Study Setting above), until the sample numbers were reached. The sample size was constrained by available resources to c.100 per community. A random, quota sampling framework was used to match the survey sample to the national 2001 census profile for age, gender, and socio-economic group (SEG) for each of the areas sampled. Response rates were between 60 and 70%.

### Ethics

This research was carried out in accordance with the Edinburgh College of Art, University of Edinburgh Ethics Board with written informed consent required from all subjects prior to taking part in the study.

### Survey variables and outcome measures

#### Demographics

Participants' ethnicity, age, and sex were recorded, together with type of housing tenure, education, relationship status (married, cohabiting with partner, single, etc.) number of children and private car access.

#### Area-level deprivation and individual socio-economic status

Area-level socio-economic deprivation was based on an independent measure—the Carstairs Index for population data in 2001 (Carstairs and Morris, 1991)—obtained via each participant's post-code. Individual socio-economic status was measured via responses to questions on education level and income coping difficulties.

#### Stress coping scenarios

Participants were asked if they felt the need to escape stress and “clear the head” on a 5-item Likert scale from *all the time* to *never*. Each participant in the survey was then asked to select one of two behavioral options they would use to escape stress or “clear the head” in the current residential environment. The behavior options were generated via qualitative methods (see section Stress Coping Scenarios above) and were presented in a two-stage process. Firstly, two environmental coping strategies were offered: an escape place within “*your own home*” or “*some other place” outwith the home*. If respondents answered “*some other place*” they were directed to three further choices: “*seeking peace and quiet*,” “*going for a walk*”, or “*seeking company*,” resulting in four coping behaviors overall.

#### Individual health and well-being variables

Our primary outcome measure of health was:

- *Perceived Stress*: measured using the Perceived Stress Scale (PSS, Cohen et al., 1983). The PSS comprises 10 items measured on a 5-point Likert scale from *never* to *very often*. The final score assesses perceived stress over the preceding month and can range from 0 (minimum level of stress) to 40 (maximum level of stress).

Secondary outcome measures of individual health were:

- *Perceived mental well-being*: measured using the Shorter Warwick-Edinburgh Mental Well-being Scale (SWEMWBS) (Stewart-Brown et al., 2009). SWEMWBS asks participants how they have felt over the previous 4 weeks in relation to 7 items used to measure aspects of mental well-being (e.g., feeling relaxed, feeling useful), with responses rated on a 5-point Likert scale from *none of the time* to *all of the time*. Final scores can range from 7 (low well-being) to 35 (high well-being).
-*Perceived general health*: measured via a single item asking participants to rate their general health, ranked on a 5-point Likert scale from 1 (*very poor health*) to 5 (*very good health*).
- *Self-reported physical activity levels:* measured using one item asking for the number of days on which physical activity (of sufficient exertion to raise breathing rate) reached or exceeded 30 min, recalled over the past 4 weeks. This item is recommended by the British Heart Foundation National Centre (Milton et al., 2011).
- *Social well-being:* measured using three items: place belonging, (“how strongly do you feel you belong to your neighborhood or local area?”) ranked on a 5-item scale from *strongly disagree* to *strongly agree*; social isolation (“how often do you feel that you lack companionship?”), ranked on a 3-item scale of *often, some of the time* or *hardly ever*; and neighborhood trust (how comfortable are you giving your home key to a neighbor to keep an eye on while you are on holiday), ranked on a 4-item scale from *very uncomfortable* to *very comfortable* (Ward Thompson et al., 2016).

#### Place characteristics

- Perceptions of green space access, quantity and quality:
  Perceived quality of local green space was measured using three items (i.e., safety, attractiveness, satisfaction with quality), ranked on a 5-item Likert scale from *low* (1) to *high* (5). Distance to local green space was measured on 5-item scale, with codes 1 to 4 indicating walking distance [from *less than 5 mins* (coded 1) to *more than 30 min walk away* (coded 4)] and 5 indicating *don't know*. In addition, we included a question to capture contact with nature from the home, *access to a garden* (labeled yes/no) and *a view to green space* (labeled yes/no).
- Quantity of green space:
  Objective measure: The quantity measure used in the analysis is a datazone green space measure based on reclassifications of the Ordnance Survey MasterMap and a city-wide audit of greenspace for Edinburgh, using classifications under Scottish Government's 2008 Planning Advice Note on Planning and Open Space (The Scottish Government, 2008) and cross-referencing to Scotland's Greenspace Map (Greenspace Scotland, 2011). The analysis carried out on green space quantity is based on more recent mapping and verification of land use data (post-2008) than the census ward level data used for case study selection (see section Study Setting), and is at a finer spatial resolution. The *percentage green space area* derived by this means included public green space, private gardens, and other green space, such as roadside trees and grass, but did not include woodland or forestry areas that were publicly inaccessible. Further information on green space characteristics can be found in Ward Thompson et al. (2016).
  Subjective measure: In addition, we asked two questions on the perceived quantity of green space in the neighborhood, the first measuring levels of green space on a 4-item Likert scale from *low* to *high*, the second measuring whether there was “sufficient green space in the neighborhood,” ranked on a 5-item Likert scale from *no definitely not* to *yes, definitely*.
- Motivation for visiting green space: We asked one question about motivational drivers for visiting local green space, with 7 options linked to known pathways linking green space with health: visiting for relaxation/peace and quiet; to get fresh air; to see wildlife/birds; for social interactions and activities (e.g., to play with grandchildren); for exercise (e.g., walking, cycling, jogging) or for “some other reason” with an open-ended response option.

### Approach to statistical analyses

In order to identify health sub-groups in our sample we used LCA, version 5.1, a method that we have applied previously to establish distinct sub group behaviors, for example in relation to the use of open space and childhood experiences of nature (Ward Thompson et al., 2004, 2005). The advantage of LCA is that it identifies hidden subgroup structures i.e. it will detect patterns in a sample that are otherwise unobservable, and is not limited by prior structuring or preconceptions of groupings (see Aspinall, 2007 for a description of LCA and its application in environmental research). It is widely used in social science and medical research to identify important subgroups that would not otherwise be revealed and to better target interventions.

Two approaches are available within LCA, in which either one or three steps can be used. We opted for the three step approach where:

- First a latent class model is built for a set of indicator variables. Step 1 involves selecting the right indicators and number of clusters that establish the best-fit model. At Step 1 the Bayesian information criterion (BIC) for model selection is used to determine the best fit model. A lower BIC figure indicates a better model fit.
- Cases are assigned to latent classes, and this classification information is saved to a file; next LCA obtains predictions for class membership based on responses for each indicator (step 2).
- In the third step the latent classification scores saved in step 2 are related to further variables of interest e.g., environmental variables.

The three step approach is preferred according to Bakk et al. (2014) since it involves first building a latent class model and then relating it to covariates or distal outcomes. However, until recently the 3 step approach has been biased in underestimating parameter estimates in the 3rd step. The method we have used follows work by Bakk et al. (2014) in correcting for bias in this third step.

Latent class has a number of advantages, including being able to better manage variables of mixed measurement type. In all cases, latent class takes any variable (e.g., categorical or continuous) and divides it into the most evenly based categories it can find, although the frequency numbers in each category are unlikely to be exactly the same. This can generate fewer categories for some variables than allowed for in the ordinal Likert scale metrics described above e.g., LCA collapsed general health into three categories to equalize numbers in each of the ordinal scale categories; these categories are shown in parenthesis in **Table 4**.

## Results

Descriptive statistics for individual characteristics of the sample can be found in Table 1, and for the environmental variables in Table 2. Table 1 confirms that our sample is very economically deprived, with 31% finding it difficult to cope on current income and with a Carstairs Index range from 3.7 to 8.7 (mean = 6.15, SD = 2.36), meaning that *all* of the sample is within the top 11% most deprived post-code areas in Scotland, according to this index.

**Table 1:** Descriptive statistics for individual variables, *n* = 406.

		**Percentage sample**	**Mean (SD)**
**Demographics**	Mean age		44 (17.1)
	16–34	34.6%	
	35–54	36.3%	
	55–64	11.6%	
	64+	17.5%	
	Gender (M = male, F = female)	M = 45%	
		F = 55%	
	Education level (% tertiary+)	14.5%	
	No of children (yes)	40%	
**Socio-economic**	Level of deprivation (Carstairs Index)		6.15 (2.36)
	Income coping: finding it “difficult/very difficult” on present income	31%	
	Car access, % “yes”	39.5%	
**Health and wellbeing**	Need to escape stress: yes “*quite often/all of the time*”	40.4%	
	Perceived stress (PSS)		15.37 (6.02)
	Perceived wellbeing (SWEMWBS)		25.35 (5.02)
	Reported physical activity (days/month)		10.32 (10.11)
	Perceived general health		3.9 (1)
**Social wellbeing**	Place belonging (score)		3.91 (0.85)
	Neighborhood trust (score)		2.90 (0.97)
	Social isolation (score)		2.51 (0.63)

*Stress (PSS) scores: higher value, greater stress; for all other health variables (e.g., general health; social isolation): a higher value, a better outcome; for level of deprivation a higher score, higher poverty*.

**Table 2:** Descriptive statistics for environmental variables.

	**Percentage sample**	**Total mean (SD)**
Average percentage GS (objective measure) in the n/hood	56.83% (SD = 12.34)	
GS satisfaction with quality		3.63 (0.78)
GS attractiveness		3.62 (0.74)
GS distance		4.33 (0.51)
Access to a garden: percentage reporting “yes”	49%	
View to GS from Home; percentage reporting “yes”	69%	

*On all green space measures, a higher score = higher satisfaction/attractiveness/closer distance to green space*.

### Descriptive statistics for LCA covariates

#### Step 1: Identifying the different health clusters across age groups (RQ1)

The main indicator entered into the health cluster model was perceived stress (PSS), alongside two further self-report indicators of health: general health and well-being (SWEMWBS). At an early exploratory stage there was found to be a significant interaction between general health and age, resulting in the latter being added as an indicator in the basic health model.

Applying the Bayesian Information Criterion (BIC) criteria for model selection (i.e., a lower value indicates better model fit), a 3 cluster model was selected (BIC value 3555.42, Table of Results provided in Supplementary Information). In addition, while for 3 clusters the *p*-value is significant, as a follow-up check, the bootstrap Chi Square *p*-value (as a more reliable estimate) showed a *p*-value of 0.174; therefore the model is a good fit. All bivariate residuals were <1.0 having adjusted the age-health interaction. The 3-class model was therefore selected as optimal.

*Predictors of class membership*: Three health indicators (i.e., perceived stress, general health, subjective well-being) and age are all highly significant in discriminating between the 3 clusters, as shown by the *p*-values in Table 3 below. The table shows the significance of the parameter estimates. The R squared value indicates how much variance of each indicator is explained by the cluster model (i.e., the extremes being 62% of well-being and 18% of general health).

**Table 3:** Parameter estimates for 3 class LC model.

**Models for indicators**				
	**Cluster 1**	**Cluster 2**	**Cluster 3**	**Wald**	***P*****-value**	**R2**
Age	−0.04	0.06	−0.02	11.53	0.00	0.34
General health (GH)	0.70	−0.12	−0.58	15.85	0.00	0.18
PSS	−0.06	−0.14	0.20	32.63	0.00	0.31
SWEMWBS	0.38	0.14	−0.53	15.58	0.00	0.62
Interaction effect^*^						
**GH**	**Age**	**Wald**	***P*****-value**			
	−0.02	12.56	0.00			

* *There is a significant interaction effect between general health (GH) and health cluster membership*.

#### Step 2: Predictions for cluster membership

Table 4 below shows the probability of an indicator variable score or range given cluster membership. The Table shows (in the first row) that 39% of the sample are in Cluster 1, 33% are in Cluster 2 and 29% are in Cluster 3.

**Table 4:** Probability of indicator variable given cluster membership.

	**Cluster 1**	**Cluster 2**	**Cluster 3**
	***Low stress Youth***	***Low stress Seniors***	***High stress Mid-age***
**Cluster size**	0.39	0.33	0.29
Indicator (LCA coding in parenthesis)
**Perceived Stress (PSS)**			
Very low PSS (1–9)	0.22	0.36	0.01
Low PSS (10–13)	0.25	0.27	0.06
Average PSS (14–15)	0.24	0.20	0.15
High PSS (17–19)	0.18	0.12	0.23
Very high PSS (20–31)	0.11	0.05	0.55
Mean PSS *(higher score indicates higher stress)*	12.9	10.7	19.0
**General health (GH)**			
Very poor to average GH (1–3)	0.02	0.26	0.31
Good general health (4)	0.39	0.52	0.49
Very good GH (5)	0.59	0.22	0.20
Mean GH *(higher score indicates higher GH)*	12.9	10.7	18.9
**Subjective Wellbeing (SWEMWBS)**			
Very low SWEMWBS (1–10)	0.00	0.01	0.66
Low SWEMWBS (11–14)	0.08	0.23	0.26
Average SWEMWBS (15–16)	0.28	0.38	0.06
High SWEMWBS (17–18)	0.25	0.23	0.02
Very high SWEMWBS (19–23)	0.39	0.15	0.00
Mean SWEMWBS *(higher score indicates higher wellbeing)*	29.8	28	21
**Age**			
16–25 (1–10)	0.36	0.02	0.19
26–36 (11–21)	0.28	0.06	0.22
37–47(22–32)	0.22	0.15	0.26
48–63 (33–46)	0.11	0.29	0.21
64–87 (47–63)	0.03	0.48	0.12
Mean age	34	57	41

*The columns under each indicator add up to 1. This is interpreted as the probability of an individual being in a particular indicator range given they are in a particular cluster*.

The values under the cluster columns are the probabilities of being in a health or age category given a person is in Cluster 1, 2, or 3. For example, given a person is in Cluster 1, the probability of being in the “*very high/high stress” category is 0.29 or 29);* by contrast, given a person is in Cluster 3, the probability of being in this high stress category is 0.79 or 79%.

Based on these data, we have labeled the clusters as follows:

Cluster 1: “*Low-stress Youth*” characterized by young adults (63% aged 16 to 36) experiencing relatively low stress, high well-being, in good general health.
Cluster 2: “*Low-stress Seniors*” characterized by older people (47% aged 64 to 87), experiencing low stress but in poorer general health and with lower well-being.
Cluster 3: “*High-stress Mid-Age*” characterized by young to middle aged adults (47% aged 26 to 47), experiencing high stress, poor well-being, and poor general health.

**Figure 1:**
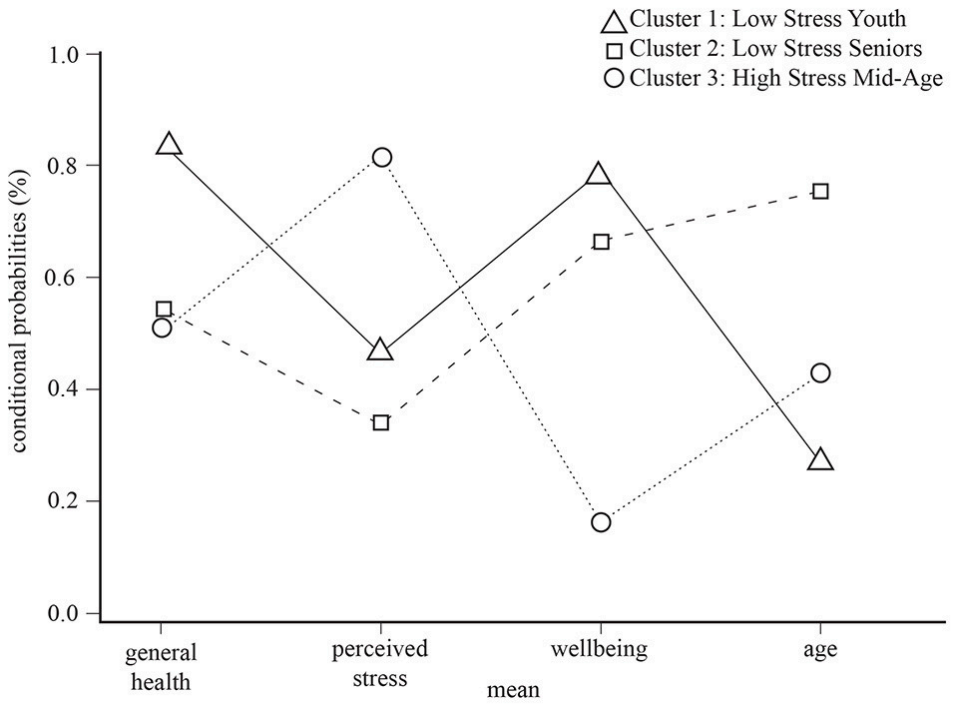
Stress cluster membership plot.

Figure 1 illustrates each cluster diagrammatically; it pictures the profile table above. Cluster 1 and Cluster 3 have an orthogonal, diametrically opposed pattern, but are closest in age.

#### Step 3: Associations between class membership and covariates

Using the three-step LCA approach next, we used linear regression to regress a series of covariates on class membership. The covariates entered into the model included, first, stress coping scenario, second, demographic/socio-economic variables, and other individual indicators (i.e., self-reported physical activity levels and social well-being) and, third, place characteristics—including urban green space—as described in section Methods above.

**Figure 2:**
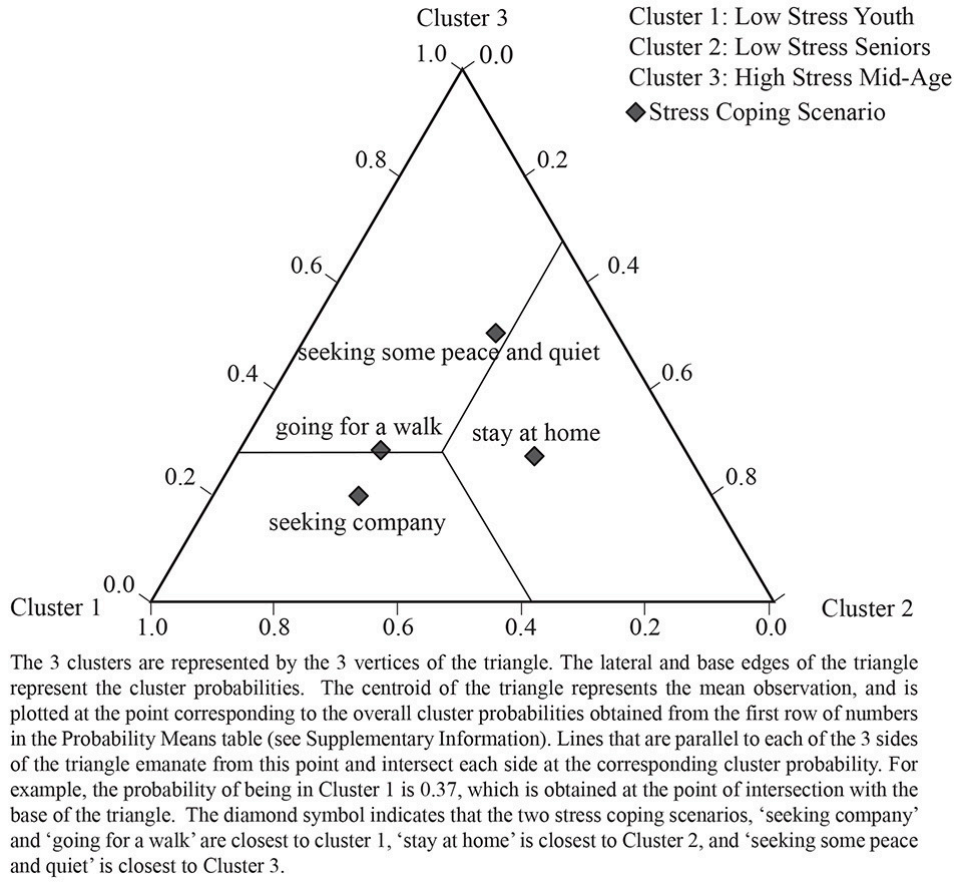
LCA tri-plot showing relationships between health cluster and stress coping scenario.

Tables 5, 7 show the significance of the parameter estimates for each covariate, and Tables 6, 8 show the probability of an indicator variable given a person is in Cluster 1, 2, or 3 (further explanatory notes on reading these tables is provided in the appropriate sections below). LCA also provides diagrammatic data in the form of tri-plots which plot the probability of cluster membership given an indicator variable (the inverse of the above). Each vertex of a triangle represents one of the 3 clusters in the LCA model. The data for these plots are provided in Supplementary Information. See Figure 2 for a full explanation on how to interpret the LCA tri-plot.

**Table 5:** Significance of parameter estimates for individual covariates.

**Indicator**	**Cluster 1**	**Cluster 2**	**Cluster 3**	**Wald**	***p*-value**
	***Low stress Youth***	***Low stress Seniors***	***High stress Mid-age***		
**Stress coping scenario**					
Staying at home	−1.78	2.75	−0.98	19.02	0.004
Seeking company	1.36	−1.31	−0.05		
Seeking peace and quiet	−0.19	−0.37	0.56		
Going for a walk	0.61	−1.08	0.47		
**Gender**					
Male	−0.47	0.82	−0.35	6.42	0.04
Female	0.47	−0.82	0.35		
**Income coping**					
Very difficult	−1.10	−2.73	3.82	15.52	0.017
Difficult	−0.24	−0.74	0.98		
Coping	1.94	0.67	−2.61		
Comfortable	−0.60	2.80	−2.20		
**Housing Tenure**					
Private landlord	3.53	−3.72	0.20	18.20	0.02
Social landlord	3.39	−1.42	−1.96		
Mortgage/shared T	2.35	−0.25	−2.10		
Owner outright	−21.98	14.68	7.30		
Neither/don't pay	12.72	−9.30	−3.42		
**Disability**					
Yes	−2.00	0.92	1.09	8.76	0.02
No	2.00	−0.92	−1.09		
**Number of children**					
Yes	2.79	−3.82	1.04	5.11	0.07
No	−2.79	3.82	−1.04		
**Car access**					
Yes	0.53	0.65	−1.18	13.52	0.001
No	−0.53	−0.65	1.18		
**Carstairs deprivation index**	−0.31	0.84	−0.53	13.61	0.001
Neighborhood trust	0.23	−0.62	0.34	5.41	0.06
Place belonging	−0.20	1.34	−1.15	8.50	0.01
Social isolation	0.07	1.63	−1.70	12.21	0.002
Physical activity	0.18	−0.16	−0.03	19.02	0.002

**Table 6:** Probability of individual indicator variable given cluster membership.

		**Cluster 1**	**Cluster 2**	**Cluster 3**
		***Low stress Youth***	***Low stress Seniors***	***High stress Mid-age***
Cluster size		0.37	0.35	0.28
Stress coping scenario	Staying at home	0.32	0.65	0.50
	Seeking company	0.42	0.18	0.18
	Seeking peace and quiet	0.03	0.08	0.17
	Going for a walk	0.23	0.09	0.15
Gender	Male	0.41	0.52	0.40
	Female	0.59	0.48	0.60
Income coping	V Difficult	0.02	0.01	0.19
	Difficult	0.35	0.15	0.33
	Coping	0.52	0.62	0.29
	Comfortable	0.10	0.20	0.12
Carstairs deprivation index	1–4	0.07	0.02	0.06
	5–5	0.22	0.42	0.47
	6–7	0.32	0.33	0.17
	8–8	0.38	0.22	0.30
Housing tenure	Rental: private	0.16	0.04	0.17
	Rental: social	0.66	0.56	0.63
	Mortgage/shared tenure	0.14	0.16	0.09
	Home owner	0.00	0.21	0.04
	Rent-free	0.03	0.00	0.01
Disability	Yes	0.03	0.14	0.10
	No	0.95	0.80	0.82
Children	Yes	0.56	0.05	0.44
	No	0.37	0.90	0.49
Car access	Yes	0.41	0.56	0.29
	No	0.57	0.43	0.68
Physical activity (days/month)	1–1	0.07	0.33	0.21
	2–10	0.10	0.16	0.34
	11–14	0.25	0.22	0.15
	15–21	0.27	0.15	0.12
	22–25	0.30	0.13	0.19
Neighborhood trust	v. uncomfortable	0.18	0.11	0.18
	fairly uncomfortable	0.15	0.10	0.14
	fairly comfortable	0.41	0.37	0.36
	comfortable	0.25	0.39	0.30
Place belonging	strongly disagree	0.00	0.00	0.04
	disagree	0.05	0.01	0.10
	neither disagree/agree	0.09	0.04	0.14
	agree	0.63	0.50	0.53
	strongly agree	0.21	0.44	0.18
Social isolation	often	0.01	0.07	0.16
	some of the time	0.16	0.24	0.42
	never	0.82	0.68	0.41

*The figure is interpreted as the probability of an individual being in a particular indicator range given they are in a particular cluster*.

**Table 7:** Significance of parameter estimates for environmental covariates.

**Indicator**	**Cluster 1: *Low stress Youth***	**Cluster 2: *Low stress Seniors***	**Cluster 3: *High stress Mid-age***	**Wald**	***p*-value**
GS visits summer	0.93	−1.40	0.47	7.97	0.02
GS distance	−1.08	−2.92	4.01	5.19	0.07
GS quantity: objective measure	−0.08	0.08	0.00	5.32	0.07
Access to garden					
Yes	−1.60	1.84	−0.24	7.00	0.001
No	1.60	−1.84	0.24		
View from home					
No	2.02	−1.82	−0.21	14.91	0.001
Yes	−2.02	1.82	0.21		
Satisfaction with quality of GS	−1.34	1.25	0.09	12.84	0.002

**Table 8:** Probability of green space indicator variable given cluster membership.

**Indicator**		**Cluster 1: *Low stress Youth***	**Cluster 2: *Low stress Seniors***	**Cluster 3: *High stress Mid-age***
GS visits in summer months	Never	0.17	0.26	0.13
	Once a year	0.02	0.17	0.02
	Once a month	0.13	0.14	0.10
	Once/week	0.34	0.26	0.36
	Everyday	0.31	0.17	0.29
GS distance from home [minutes (m) walking]	>30 m walk	0.00	0.01	0.00
	15–30 m walk	0.02	0.03	0.01
	5–15 m walk	0.75	0.75	0.43
	<5 m	0.20	0.21	0.48
GS quantity: percentage	1–7 (<33%)	0.23	9	8
	8–12 (34–49%)	0.19	0.14	0.14
	13–17 (50–58%)	0.19	0.12	0.22
	18–22 (59–62%)	0.07	0.21	0.13
	23–29 (>63%)	0.09	0.23	0.14
GS access to garden	Yes	0.24	0.61	0.38
	No	0.75	0.39	0.61
GS view from home	Yes	0.09	0.32	0.37
	No	0.91	0.68	0.62
Satisfaction with quality of GS	1–3 (low quality)	0.29	0.18	0.48
	4–4 (high quality)	0.49	0.65	0.40
	5–5 (very high quality)	0.07	0.13	0.05

### Associations between latent health clusters and stress coping scenarios (RQ2)

Our second research question concerned the potential behavioral choices taken to escape stress and their possible association with different health clusters. Table 5 shows the LCA regression output and shows that cluster membership is statistically distinguished by the four stress coping scenarios to a highly significant level (*p* < 0.004). Table 6 shows the probability of cluster membership according to these four stress coping scenarios.

We can see from Table 6 that:

***Low-stress Youth*** are more likely to escape stress by “*seeking company*” (42%) or “*going for a walk*” (23%).
***Low-stress Seniors*** are most likely to escape stress by “staying at home” (65%).
***High-stress Mid-age*** people are most likely to escape stress by “*staying at home*” (50%) but—in our sample—also have the highest probability of “*seeking peace and quiet*” (16%) away from home.

Figure 2 plots the probability of being in a health cluster given one of four stress coping scenarios and shows, for instance, the option “*staying at home*” is closest to Cluster 2, *Low-stress Seniors*.

### Associations between latent health clusters and individual characteristics (RQ3)

Our third research question addressed how area-level deprivation and individual characteristics, including social well-being and physical activity levels, are associated with different health clusters. Table 5 also shows the LCA regression output for these variables across the three latent health clusters.

Table 5 shows that a number of demographic/social-economic variables (i.e., gender, disability, children, deprivation, tenure, subjective income coping and car access) distinguish between the three latent health clusters; of these, car access and deprivation score were the most significant discriminators (*p* < 0.001). Table 6 shows the probability of cluster membership according to these individual discriminators, described below:

***Low-stress Youth*** are more likely to be female (59% probability), living in the most deprived neighborhoods (70% in upper deprivation categories), renting from a social landlord (66%), with an average chance of coping well on a low income (52%), quite likely to have children under 16 (56%); with a low chance of experiencing a disability (3%) and of having a car (41%).
***Low-stress Seniors*** are marginally more likely to be male (52% probability), experiencing high level deprivation (55% in upper deprivation categories), renting from a social landlord (56%), but also more likely than in other groups to be a home owner/have a mortgage (37%); likely to be coping better on a low income (62%), with a very high likelihood of having children (90%), a low likelihood of disability (14%), and higher likelihood of having a car (56%).
***High-stress Mid-age*** people are more likely to be female (60%), with an average chance of finding it *very difficult/difficult* to cope on a low income (52%), renting from either a social landlord (63%) or private landlord (19%); likely not to have a car (68%), unlikely to have a disability (10%), with a lower probability of having children than in other groups (44%).

Our third research question also asked how other individual characteristics—including social well-being—are associated with different health clusters. Table 5 shows that both physical activity levels and social well-being (i.e., place belonging and loneliness) significantly discriminate between the latent health clusters. Table 6 shows the probability of cluster membership according to social well-being and physical activity:

***Low-stress Youth*** are more likely to be physically active on regular basis (57% in the upper activity categories) and very likely to have good social well-being.
***Low-stress Senior***s are less likely to be physically active (49% in the lower activity categories) but likely to have good social well-being.
***High-stress Mid-age*** people are likely to be physically *inactive* (55% in the lower activity categories) and experience poor social well-being.

### Associations between latent health classes and environmental characteristics (RQ4)

Table 7 shows the LCA regression output and statistically significant environmental predictors of the three latent health clusters. Table 8 shows the probability of cluster membership based on the above predictors. Cluster membership by environmental characteristics can be described as follows:

***Low-stress Youth*** characterized by good access to and good use of local green space, and reasonable satisfaction ratings. The probability of good access to local green space is high: the probability of living “*within a 5–15 min walk” is 74%*, the probability of visiting green space “*at least once a week/every day in summer*” is 64% and the chance of being *very satisfied/satisfied* with the quality of local green space is 56%. The probability of having a view from home and/or a garden is low.
***Low-stress Seniors*** characterized by good access to a garden and local green space, but infrequent use. The probability of having a garden is 61%; the probability of living close to green space is also high (a 75% chance of living “*within a 5–15 min walk*”) but the probability of visiting that green space frequently is relatively low (43% probability of visiting “*at least once a week/every day in summer*”) despite a high probability of being “*very satisfied/satisfied*” with local green space (78%).
***High-stress Mid-age*** characterized by good access to local green space, reasonable use but poor satisfaction ratings. The probability of having good access to green space is very high (90% chance of being “*within a 5–15 min walk/less than 5 min walk*”); likely to visit green space fairly regularly in summer (65% probability of visiting “*at least once a week/every day in summer*”), but less likely to be satisfied with it (48% in lowest satisfaction categories). The probability of having a view from home and/or a garden is low.

**Figure 3:**
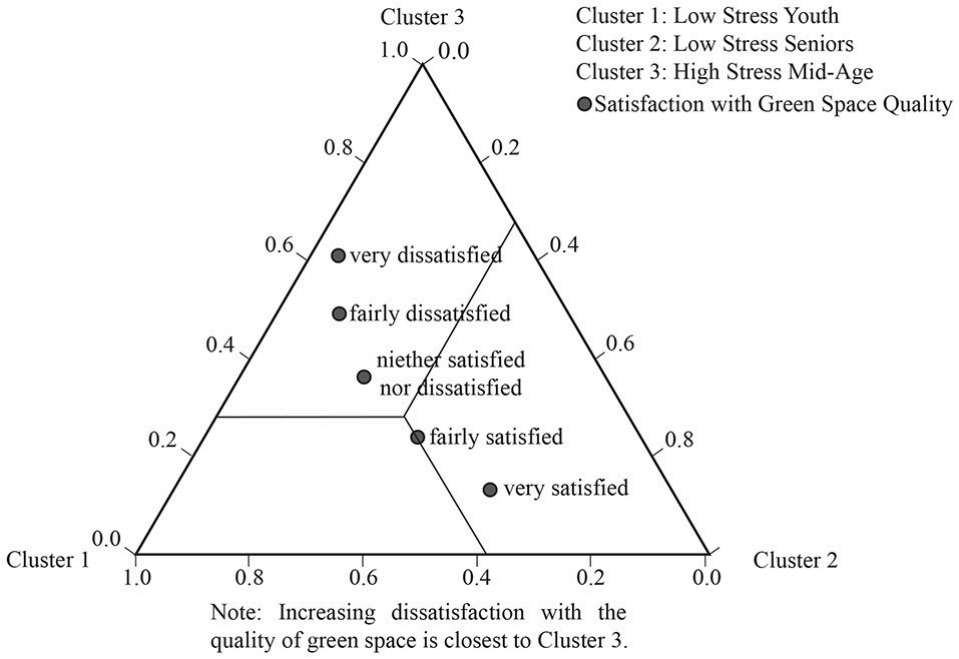
LCA tri-plot showing relationships between green space satisfaction and the heath clusters.

**Figure 4:**
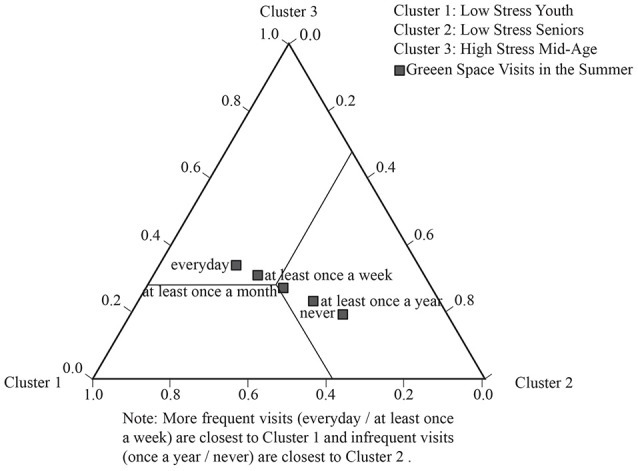
LCA tri-plot showing relationships between green space visits (summer) and the heath clusters.

**Figure 5:**
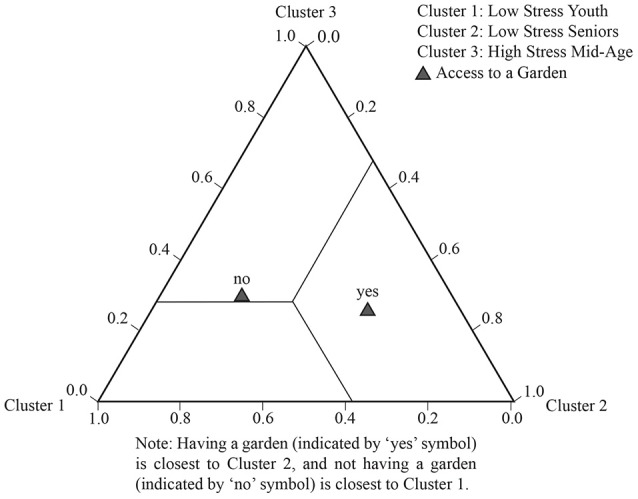
LCA tri-plot showing relationships between garden access and the heath clusters.

Three LCA tri-plots (Figures 3–**5**) illustrate the strongest patterns between the environmental variables and health clusters (i.e., the most significant environmental discriminators); these diagrammatically plot the probability means from the LCA output (data in Supplementary Information).

The tri-plot in Figure 3 shows increasing dissatisfaction with green space goes with increasing stress.

On use of green space (in summer) (a significant discriminator of health clusters at *p* = 0.02), we see a strong association with age (see Figure 4), with more frequent visits closest to the health clusters characterized by youth and mid age (Clusters 1 and 3, respectively) and tailing off to no visits in *Low-stress Seniors* (Cluster 2). The LCA probability means (which the triangle illustrates) show an interesting difference in the young-mid-age clusters, with the probability of visiting *every day/at least once a week* high in *Low-stress Youth* (Cluster 1) (85%), falling off in *High-stress/Mid-age* (Cluster 3) to 64% (data in Supplementary Information).

Another highly significant predictor variable, contact with nature via garden access (*p* < 0.001) is highest in *Low-stress Senior*s Cluster (2), with access clustering in this older age category (a 52% probability of having a garden). This pattern is illustrated in Figure 5.

### Motivations for visiting green space by health cluster

**Figure 6:**
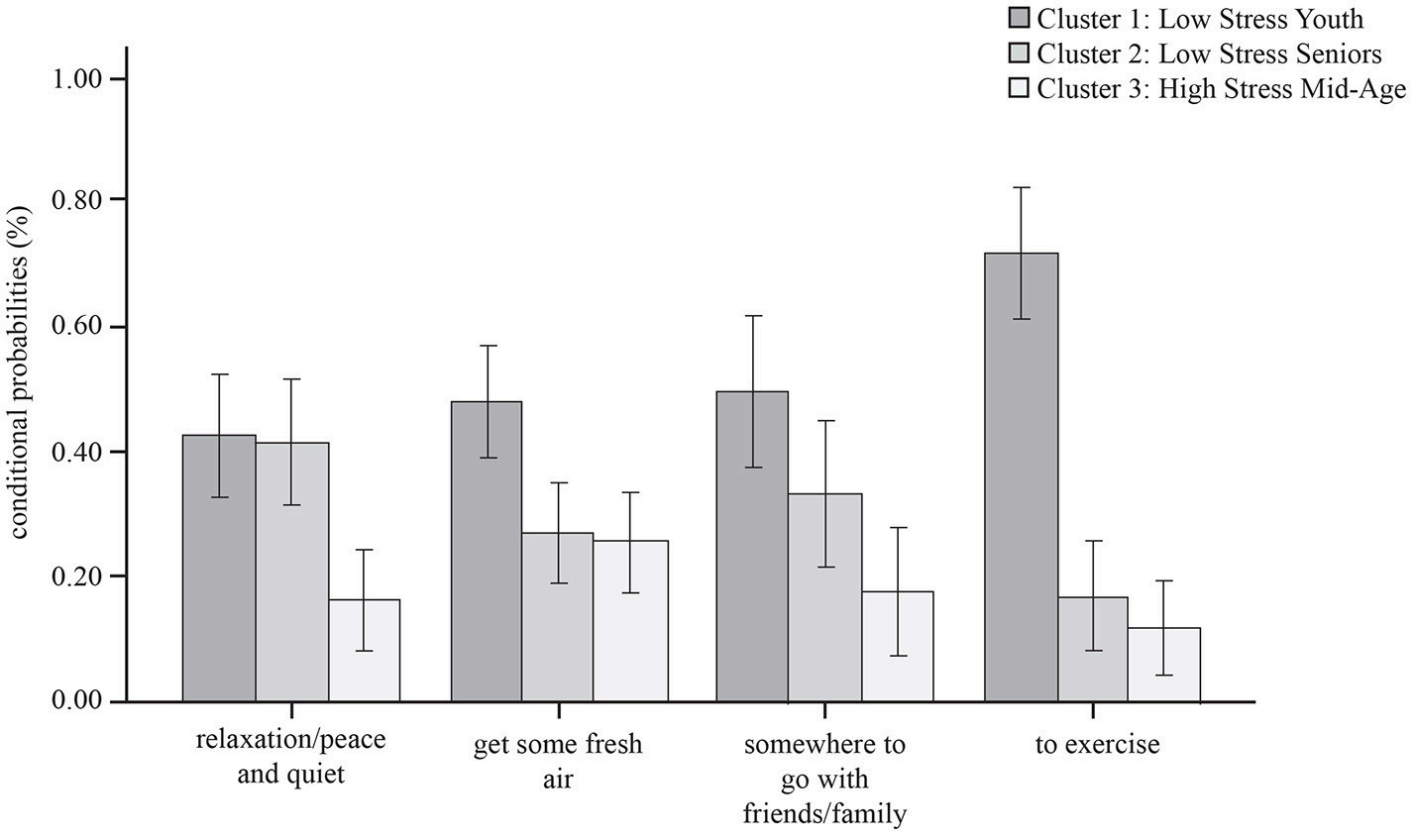
Primary motivations for visiting local green space by health cluster.

The motivations for visiting urban green space significantly vary by health cluster. Figure 6 shows that *Low-stress Youth* (Cluster 1) are more likely to visit for exercise and for social reasons (possibly reflecting their chosen stress relief behaviors to walk and seek company). Both *Low-stress Youth* (Cluster 1) and *Low-stress Seniors* (Cluster 2) are equally likely to visit for relaxation. *High-stress Mid-age* (Cluster 3) show distinctly different motivational patterns and are much less likely to visit urban green space for relaxation, exercise or social purposes.

We found no significant patterns of difference across the three health clusters in social visitation patterns (i.e., going alone or with a friend).

### Summary of findings

LCA identified three latent health clusters:

1. ***Low-stress Youth*** characterized by young people, most likely to be aged 16 to 24, who seek company and walk as their preferred stress coping scenarios, are physically active, with good social well-being, have good access to green space, regularly visiting these spaces in summer, and are satisfied with green space quality.
2. ***Low-stress Seniors*** characterized by older people aged 65+, who stay at home as their preferred stress coping scenario, are in poor general health, with good social well-being, but relatively physically *inactive*, infrequently visiting local green space (despite good access) but likely to have contact with nature via good access to a garden, which may be offering some buffer to life stressors. Despite good access to other green space, perceived as high quality, this stress cluster is not using public open space for stress regulation.
3. ***High-stress Mid-age*** characterized by young-middle adulthood, most likely to be aged 25 to 47, who stay at home as their preferred stress management scenario, in poor general health, with poor social well-being, physically *inactive*, frequently visiting local green space (in summer) but most likely to be dissatisfied with its quality. Despite good access to green space, this stress cluster is not using public open space for physical activity or stress relief; we suggest this is likely owing to perceptions of its poorer quality.

## Discussion

Following our earlier study on stress mitigation in the same deprived urban population (Ward Thompson et al., 2016), our interest in the current study was in *how* the local environment (including one's home) might assist with stress regulation in a population experiencing higher than average stress. Whilst our earlier study established a relationship between perceived stress and access to green space (including quantity of green space in the neighborhood and access to a garden/allotment), it did not establish *why* green space has this effect. For instance, is the relationship owing to people being more physically active or more social in their local green space, or both? Whilst physical activity, mental relaxation, and social interactions are believed to be potential pathways to the health benefits of green space (Lachowycz and Jones, 2013; Hartig et al., 2014), current research evidence in deprived urban populations is very limited. The aim, therefore, in the current study was to tease out *how* people use their immediate and local environment for stress regulation, and how these behaviors relate to perceived stress.

Firstly, we used LCA to identify health sub groups in a population experiencing economic stress. The best fit model was a three cluster model with perceived stress and age the most significant discriminators (RQ1). Our youngest participants (likely to be aged 16–24) were the healthiest; our mid-age participants (likely to be aged 26 to 47) were the least healthy; our older participants (likely to be aged 64 to 87 group) were unhealthy, but the least stressed of our sample.

Secondly, LCA established relationships between health cluster membership and a series of indicator variables (or co-variates). First, it established a relationship between health membership and people's hypothetical stress coping strategies. Our healthiest cluster (*Low-stress Youth*) are most likely to seek company (outside of the home) and walk to escape stress; they are more physically active, in better general health, and have better subjective and social well-being. By contrast, our poorest health cluster (*High-stress Mid-age*) is more likely to stay at home for stress relief, as is Cluster 2 (*Low-stress Seniors*). These two health clusters have lower physical activity levels and poorer general health. *Low-stress Seniors*, however, are the most robust to stress in our sample: it is possible that this health cluster is experiencing sensory contact with nature via greater access to a garden and/or a view from home, and that this is helping buffer stress levels.

Next, LCA established relationships between health cluster membership and individual characteristics including gender, subjective income coping, housing tenure, and deprivation indices (RQ3). For instance, health clusters are characterized by gender differences: younger to mid-aged women (aged 48–62) were the most stressed in our sample (Cluster 3, *High stress Mid-age*) (Table 6). We also found strong relationships between health clusters and income coping: the probability of coping on a low income is significantly lower in *High stress Mid-age* (29%), as compared to the other (better) health groups (Table 6). Raising children had marginal significance on health membership (*p* = 0.07). Whilst raising children is an identified stressor in families living with poverty in the US (Kuo, 2001), we found the inverse pattern in our sample. Our younger, healthier group are more likely to be raising children under the age of 16 (Table 6) than less healthy sub-groups. We suggest having children might therefore be acting as a moderator in the relationship between getting outdoors more frequently and being more social. For instance, parents/carers with children are more likely to be walking outdoors to and from routes to school and interacting with eachother on a daily basis.

Finally: we found that health cluster membership is strongly related to a range of green space attributes (RQ4). *Low-stress Youth* are most likely to be satisfied with their local green space, have good access, and have a higher likelihood of visiting their local green space in summer (this pattern also continues into winter visits). They are motivated to visit local green space for a range of relaxation, exercise and social purposes. By contrast, *High-stress Mid-age* people, whilst having a similar likelihood of good access to green space, have much lower perceptions of its quality, and are less motivated to visit for relaxation, exercise or social purposes. Whilst use of green space is very low in *Low-stress Seniors*, this group has the highest likelihood of immediate contact with nature via access to a garden or a view from home, and we suggest that this visual contact to nature may offer a buffer against life stressors, although not contributing to general health or physical activity levels in our sample.

Despite good access (all three latent health clusters live reasonably close to their local green space) it appears that relationships between *use of* and *satisfaction* with urban green space is moderated by age. Whilst *Low-stress Youth* are using nearby green space regularly and are satisfied with its quality, the same (or similar) outdoor space is not supporting the needs of young-middle aged adults, most of whom simply stay at home for stress relief. As one approaches mid-life, perceptions of *quality* of the local environment may become more discerning, or this age group may demand different attributes from green space. By contrast, *Low-stress Seniors* are satisfied with the quality of their local green space but don't appear to use it. The motivations for use of local green space also vary across our three clusters, *Low-stress Youth* are most likely to visit for exercise; *Low-stress Seniors* are most likely to visit for relaxation; whilst the *High-stress Mid-age* people appear to have no strong motivations for visiting green space. In this deprived urban population, it appears quality judgements about green space are more important for utilizing green space than either the amount of green space available or proximity, but judgements clearly vary across the lifespan.

Our study's finding that quality of green space is significantly related to health group membership is an important finding since research on the health benefits of green space is largely focused on issues of quantity or proximity, using objective measures of distance to green space or percentage calculations. Addressing issues of quality of green space—and its relationship to health and well-being—is a theme identified in our prior research in deprived urban communities (CABE, 2010; Ward Thompson et al., 2013; Roe et al., 2016). Poorer communities live with both poorer access to green space and poorer quality green space (CABE, 2010). Lennon et al. (2017) argue that more attention be paid to quality, alongside issues of proximity, and for a more nuanced and dynamic understanding of green space use and perceptions. The authors suggest a framework of affordances to capture multidimensional perspectives of quality amongst diverse sub-groups (e.g., by age, gender, ethnicity). Quality is conceptualized in terms of the opportunities (or constraints) a park offers in relation to six attributes: space (e.g., landforms); scale; time; objects (e.g., presence of absence of trees, benches, cycleways); actions (e.g., climbing, jogging, bird watching); and the physical and psychological state of the person positioned in relation to these other dimensions. Understanding how these attributes interact to generate quality green space experiences is one promising area for future research.

As far as we know, this is the first study to explore health sub-groups and relationships to environment in terms of stress regulation in a deprived urban population. The current literature on environmental emotional regulation and the benefits of nature in supporting mood regulation is largely focused on younger populations such as students (e.g., Korpela et al., 2001) or less deprived populations (e.g., Korpela, 2003; Korpela and Ylén, 2007). Understanding how the neighborhood environment, including the home, supports stress regulation in people living in poverty is therefore an important contribution to this body of research. Furthermore, since there is evidence that going for a walk in local green space offers opportunities for reflection and to “think things through,” reducing negative thinking and rumination (Bratman et al., 2015), then if nature access can be increased in these communities, one important potential benefit may be a reduction in mental health inequities. Evidence shows that access to green space is associated with a reduction of up to 40% in mental well-being inequalities (Mitchell et al., 2015). We conclude that, as well as focusing on proximity and quality measures, a focus on understanding the interactions between *quality* and *use* of green space for mental well-being in people living with poverty is likely to be a fruitful approach in tackling mental health inequities.

## Implications

Although access to green space is associated with health benefits, particularly for economically deprived urban populations, the challenge in addressing health inequity is not simply about availability of green space but also how to support people living in poverty to derive health and well-being benefits from their local green space. We have shown that patterns of perception and use are likely to vary according to life stage. The advantage of LCA is that is reveals otherwise unobservable sub-groups in the population under study, and allows for interventions to be targeted at these sub-groups. To date, studies of green space proximity have rarely addressed the importance of programming and differences in lifestyle to afford greater access and benefit levels from urban green space. In applying the research findings to social and recreational policy, we suggest that *Low stress (but low health) Seniors* need encouragement to use their local green space and/or garden to improve their physical activity levels and general health. Raising awareness of the benefits of contact with nature and encouraging Seniors to be more aware, e.g., of the sensory affordances of local green space, may also have positive effects on their subjective well-being (which is low in this group). Furthermore, LCA identified mid-aged people living in poverty are at risk from high stress and poor overall health and well-being. Local initiatives to tease out why such sub-groups perceive their local urban green space as of poor quality (via focus groups and surveys) can help target physical interventions to improve the quality of local green space for health and well-being in this segment of the population. In addition, as mentioned above, better understanding of the specific park attributes that encourage use for, say exercise, or social activity, will also enhance the potential of green space for health and well-being amongst poorer health sub-groups. Finally, estimating the economic value of improving green space access for health and well-being outcomes in this demographic warrants further attention; several useful protocols have been established (Silveirinha de Oliveira et al., 2013; Wolf et al., 2015).

## Limitations

Our study was based on four hypothetical behavior choices (set within the context of the current neighborhood environment) rather than actual activities reported as undertaken for stress relief. Whilst it is reasonable to assume one's intended motives for stress reduction bear some resemblance to actual behavior, our study does not support this. For instance, the low levels of monthly reported physical activity across all health clusters indicate that most of our sample are not engaged in any regular physical activity. In future it would be important to identify *actual* stress regulation behaviors and the exact environmental context in which such behaviors take place (e.g., via mobile phone applications integrated with GPS).

Our four, proffered stress relieving activities are not mutually exclusive categories, i.e., it is possible someone “*seeking company*” away from home will also partake in the activity “*going for a walk.*” However, in asking participants to make a distinct choice between one coping activity over another, this suggests—say in the case of “*going for a walk*”—the main motivator is exercise, and that any social motivator is secondary to that intent (otherwise the participant would select “*seeking company*” as the primary activity).

A limitation of the current study is that we explored *use* and *quality* (i.e., satisfaction with the quality of green space) as single entity variables. But these concepts are multi-dimensional (i.e., *use* constitutes more than walking to and within a local green space). Two recent studies have explored interactions between use and quality perceptions of green space. The first reports that quality perceptions of open space and frequency of use of green/social spaces have a significant mediating role in the relationships between the neighborhood environment and mental well-being (Hadavi, 2017). The second study identified significant interactions between quality perceptions of specific park ingredients (i.e., satisfaction scores with the quality of different park components such as trees, lawns, flower beds etc.) and different types of use (i.e., walking, running, biking etc.) (Hadavi and Kaplan, 2016). Future research needs to better understand these active park “ingredients,” quality perceptions and their role in stress reduction.

Whilst we explored motivations for use of parks, our research on motivational affordances of local green space for stress relief was limited to one generic question presenting seven options (see section Place Characteristics, item “c”). Whilst these options were established from previous analyses of motivational patterns in similar populations, further research is needed to explore a wider range of motivations and how environmental interventions—social and physical—might shift these motivations to facilitate actions that help maintain good health.

The data for this study were collected primarily to explore differences in green space quantity and perceived stress (the subject of Ward Thompson et al., 2016). Our objective quantity measure of green space was not a significant discriminator of health clusters in this study (although marginally so at *p* > 0.07); our reporting in this study therefore does not dwell on the significance of green space differences in quantity between neighborhoods.

Finally, there is a possibility of response bias despite the quota sampling approach (see section Data Collection), in relation to characteristics not included in the quota, (e.g., people excluded because they were not at home). This was minimized by repeat household call backs by the survey company. Also, recruitment was in a deprived urban population, many of whom would be at home for various reasons (e.g., unemployment, caring for a family member).

## Conclusion

Amongst people living with high economic deprivation, our study identified three distinct health clusters and identified relationships between these health clusters and stress coping scenarios in relation to participants' local environment. Relationships were also found between health cluster membership and environmental variables, including access to urban green space and gardens. Our study has highlighted that environmental opportunities for stress regulation vary by age: younger people go outdoors more often for stress relief, appear to use green space more regularly and are less discriminating about its quality. By contrast, people in middle age experience higher levels of stress, tend to stay at home for stress relief, are more physically inactive, and more negative about the quality of their local environment. Older people are more likely to be in poor general health, and not to use their local green space, are less physically active, but happier with the quality of their local green space. We suggest policy efforts therefore focus on targeted health promotion initiatives that raise awareness of the benefits of local green space for health and well-being—but also facilitate increased access, including exploring with the local community ways in which quality can be improved.

Our study is the first to employ LCA to understand better how the local neighborhood environment—including access to local green space—affords some people opportunities for stress relief but not others. Since a significant body of experimental and epidemiological evidence now points to green space as a salutogenic and stress-mitigating environment, urban planners and designers need to engage with deprived urban communities—across the lifespan—to better understand how their local green space might better serve their health and recreational needs.

## Author contributions

All three authors conceived and designed the research. JR, as the first and corresponding author, carried out descriptive statistics and wrote up the manuscript in collaboration with the other authors. PA carried out the LCA data analyses and provided the figures and table output for the research report. CW was the Principal Investigator for the research study. All authors contributed to development of the research questions and interpretation of the findings and their implications.

### Conflict of interest statement

The authors declare that the research was conducted in the absence of any commercial or financial relationships that could be construed as a potential conflict of interest.

## Footnotes

^1^CRESH (The Centre for Research on Environment Society and Health). Available online: www.cresh.org.uk
