# Supplementary material for: Coping with Stress in Deprived Urban Neighborhoods: What Is the Role of Green Space According to Life Stage?
Source: Front Psychol. 2017 Oct 18;8:1760. doi: 10.3389/fpsyg.2017.01760 (PMC5651820; doi:10.3389/fpsyg.2017.01760)
Supplement: Supplementary file 1 [file Table1.DOCX]

Note: The lowest BIC value indicates the best-fit model, in this case a 3-cluster model

| **Table 1: Supplementary Information, Model Fit Information** | | | | | | | | |
| --- | --- | --- | --- | --- | --- | --- | --- | --- |
|  |  | LL | BIC (LL) | Npar | L2 | df | p-value | Class.Err |
| Model 1 | 1-cluster | -1785.57 | 3645.23 | 14 | 518.92 | 291 | 4.7e015 | 0.00 |
| Model 2 | 2-cluster | -1738.20 | 3585.08 | 19 | 430.16 | 286 | 7.1e-8 | 0.07 |
| Model 3 | 3-cluster | -1709.06 | **3555.41** | 24 | 371.90 | 281 | 0.00022 | 0.14 |
| Model 4 | 4-cluster | -1700.50 | 3566.88 | 29 | 354.77 | 276 | 0.00095 | 0.14 |

*Key:* LL = Log-likelihood; BIC(LL) = Bayesian information criterion based on the log-likelihood; Npar = number of estimated parameters.

L² statistic indicates the amount of the association among the variables that remains unexplained after estimating the model;
