# Supplementary material for: Coping with Stress in Deprived Urban Neighborhoods: What Is the Role of Green Space According to Life Stage?
Source: Front Psychol. 2017 Oct 18;8:1760. doi: 10.3389/fpsyg.2017.01760 (PMC5651820; doi:10.3389/fpsyg.2017.01760)
Supplement: Supplementary file 2 [file Table2.DOC]

Note, that in this table the columns reading left to right across add up to 1. This is interpreted as the probability of being in a cluster given a particular indicator category.

| **Table 2 Supplementary Information**  **Probability Means Table**  *Reports the probability of cluster membership given indicator variable and is the data plotted in the tri-plots, Figures 2 to 5.* | | **Cluster 1**  *Low stress Youth* | **Cluster 2**  *Low Stress Seniors* | **Cluster 3**  *High Stress Mid-age* |
| --- | --- | --- | --- | --- |
| **Overall probability indicators** | | 0.37 | 0.34 | 0.29 |
| **Stress Coping Scenario** | Staying at Home | 0.25 | 0.46 | 0.29 |
|  | Seeking Company | 0.58 | 0.23 | 0.19 |
|  | Seeking Peace & Quiet | 0.14 | 0.33 | 0.53 |
|  | Going for a Walk | 0.53 | 0.21 | 0.26 |
| **GS visits (summer)** | Never | 0.33 | 0.48 | 0.19 |
|  | Once a year | 0.10 | 0.81 | 0.09 |
|  | Once a month | 0.38 | 0.39 | 0.23 |
|  | At least once a week | 0.40 | 0.29 | 0.31 |
|  | Every day | 0.45 | 0.23 | 0.32 |
| **GS Acesss to Garden** | Yes | 0.22 | 0.52 | 0.26 |
|  | No | 0.48 | 0.23 | 0.29 |
| **Satisfaction with Quality of GS** | 1-3 (dissatisfied/neutral) | 0.38 | 0.12 | 0.50 |
|  | 4-4 (satisfied) | 0.41 | 0.35 | 0.24 |
|  | 5-5 (very satisfied) | 0.33 | 0.54 | 0.13 |
|  | *Mean* |  |  |  |
